# Education and Infant Welfare

**Published:** 1916-04-01

**Authors:** 


### EDUCATION AND INFANT WELFARE.

### The Views of the Board of Education on Mothercraft.

A Board of Education circular for official use has been issued which reviews the present state of voluntary and State efforts in infant care in so far as they fall within the purview of the Board. This circular consists largely of a reprint from the report for 1914 of the Board's Chief Medical Officer, with an additional appendix. It contains little or nothing that is actually novel; yet it is a distinctly valuable production in that it summarises what is being done, gives many valuable details of how it is being done, and inferentially shows what further extensions of effort the Board of Education is now prepared to sanction and to encourage.

In the first paragraph of this pamphlet a somewhat striking sentence occurs: "The environment of the infant is its mother." And although, like many catch-phrases, this epigram can easily be shown to be neither the whole truth nor nothing but the truth, at least there is a large element of truth in it. The Board of Education, says the next paragraph, is not concerned with medical, surgical, or obstetrical aid for the mother before, during, or after her confinement, nor for the provision of a sanitary environment for mother and child. But it is responsible both for bringing educational influence to bear on the problems of nurture and for the medical care, inspection, and treatment of all children of school age. It aims at combining the various agencies associated with the system of local government—educational and sanitary—in cordial co-operation with voluntary societies throughout the whole country. In this particular sphere the voluntary worker is essential to true and lasting success. This fact is recognised by the Board, and it is well that it should be. There is need for State machinery, financial assistance, and supervision; but there is also need for the spirit of neighbourly helpfulness and friendliness, organised in a simple and free manner through convenient institutions, having a minimum of "officialism" and a maximum of homeliness.

### Maternal Ignorance.

The principal operating influence, says the medical officer, with emphasis, is the ignorance of the mother, and the remedy is the education of the mother. The percentages of verminous heads and bodies among elementary school children are sufficient to prove this point, and are even clearer proofs than the figures of otorrhœa, rickets, dental caries, and other "neglect diseases" so rampant among children of school age. The four educational methods which commend themselves to the Board for the prevention of these defects and diseases are schools for mothers, day nurseries (*crèches*), nursery schools, and the training of elder girls. Incidentally, since the great majority of the girls at elementary schools some day become mothers, the most logical educational plan is the last of these four, since it ensures that girls leaving school shall at least know something (even if it be only their need for further instruction) of the care of the infants they will some day have to mother. We are glad to note that the Board of Education views such instruction for girls during their last two compulsory years at school (aged twelve to fourteen) with favour. We should be even more pleased if we were able to say that that approval develops into something less lukewarm than it seems to be at present: the circular mentions only five districts in which such classes are carried out. Rather more of this practical kind of education in the future, and rather less of the violoncello and palæontology type of the recent past, is what the nation is beginning to demand from the Board of Education.

### Schools for Mothers.

It is, however, on schools for mothers that the circular is most detailed and helpful. Seized at the same moment with the same happy thought, the Local Government Board and the Board of Education, about the middle of 1914, promulgated almost simultaneously schemes of State grants in aid for such institutions. The result was, naturally, confusion; for two authorities were propounding different schemes for very much the same sort of work. However, the two Boards have, since then, conferred together, which might have been done before; and their relative spheres are better defined in consequence of their agreement, though there must be of necessity a certain amount of overlapping between schools for mothers and maternity centres; but it is now recognised that the former are educational in intent, whereas the latter are therapeutic.

The lines laid down for the conduct of infant consultations, which are rightly regarded by the Board as a very essential part of any school for mothers, are those which have been proved in practice to work best, and are very sound. A statistical return is given of those schools for mothers which have received recognition and aid from the Board. £6,100 was paid for the year ending March 31, 1915, to 157 such schools.

Day nurseries are not dealt with at such great length in the circular, but receive more attention in the appendix. Eighty such nurseries were approved by the Board for the same year, and grants amounting to £5,100 were paid. It is pointed out that a day nursery should be associated with a school for mothers, for the two organisations tap a rather different *clientèle*: those mothers who require the one do not or cannot want the other, and *vice versa*. It is laid down that there should be in every day nursery effective supervision by a medical officer. The circular proceeds significantly that hitherto the majority of medical officers have generously given their services without payment, but that it is doubtful how long they can reasonably be expected to continue to do so, especially if they are asked to undertake increased duties. This is one out of many medico-financial problems which will probably be solved on very different lines after the war than they were before it.

